# London Healing Wells

**Published:** 1913-12-06

**Authors:** 


### London Healing Wells.

### THE PUBLIC PHARMACISTS' AND DISPENSERS' ASSOCIATION.

The second meeting of the winter session of the Public Pharmacists' and Dispensers' Association was held at St. Bride Institute, E.C., last week, when a lecture was delivered by Dr. W. Lauzun Brown on "London Healing Wells." Mr. T. H. W. Idris, J.P. (President of the Association) was in the chair, supported by Mr. R. W. Lindsey, Mr. R. Welford, Mr. W. E. Miller, Mr. W. H. Windmill, Mr. France, and Mr. Gibson. Others present included Mr. O'Brien, Mr. Jenkin, Mr. Noad Clark (Paddington Infirmary), Mr. Dunstan, Mr. Goodall, and many friends of the members. Mr. Gibbons and Mr. Moore were elected members.

The lecturer explained the means by which London obtained its water supply in the olden days—from the Walbrook and Fleet rivers—although these after a time became polluted owing to the increased population and absence of sanitary measures. A collecting-ground was then made in the vicinity of Pentonville. Eastwards from this area the chief source of water was from wells. Clerks' well (Clerkenwell) was one of the most noted, and around this at various periods fêtes were held. Here, indeed, was an early home of the drama, "miracle" plays being acted at this spot. The lecturer then mentioned the properties of the waters contained in the various wells, which apparently were a cure for all diseases. Among further examples of interest are Goswell Road, which takes its name from "Godswell"; Bridewell, after St. Bridget, the site of which was once a Royal palace; and most curious perhaps of all "Ealing," which owes its origin to the contraction of "healing" by which name this district was known. "Hanwell" is, of course, a colloquialism from "St. Anne's Well. At Muswell Hill an old well had a great reputation for the cure of skin troubles, and at one period a leper colony existed there. Bermondsey Spa, a strange conjunction to modern ears, and other noted wells were mentioned.

The lecture was illustrated with lantern slides, and proved to be full of interest to the many pharmacists and their friends present, taking, as it did, a subject not very generally discussed. Mr. Idris, in thanking the lecturer, on behalf of the Association, stated that in his opinion the public authorities' first duty was to maintain an abundant supply of pure water for its citizens.

